# Cutaneous leishmaniasis of the nose evaluated with confocal microscopy successfully treated with topical 5% imiquimod

**DOI:** 10.1016/j.jdcr.2025.10.070

**Published:** 2025-12-17

**Authors:** Antonio Di Guardo, Biagio Didona, Luca Ambrosio, Francesco Ricci, Annarita Panebianco, Giovanni Di Lella, Damiano Abeni, Luca Fania

**Affiliations:** a IDI-IRCCS, Dermatological Research Hospital, Rome, Italy; b Department of Clinical Internal, Anesthesiological and Cardiovascular Sciences, “Sapienza” University of Rome, Rome, Italy; c Department of Life Science, Health, and Health Professions, Link University of Rome, Rome, Italy

**Keywords:** cutaneous leishmaniasis, imiquimod, noninvasive imaging, reflectance confocal microscopy, skin infectious diseases

## Introduction

Cutaneous leishmaniasis (CL) is a parasitic skin infection caused by protozoa of the genus *Leishmania*, transmitted by sandflies.^1^ Cutaneous leishmaniasis remains a diagnostic and therapeutic challenge, particularly when the face is involved. Diagnosis typically relies on the detection of amastigotes in tissue smears, culture, or polymerase chain reaction-based methods. However, in many settings, these tools are either unavailable or impractical, and diagnosis remains predominantly clinical. In this context, noninvasive diagnostic methods, such as dermoscopy and reflectance confocal microscopy (RCM), are emerging as valuable tools.^2^^,^^3^ Although systemic or intralesional treatments are commonly employed, these may not always be feasible due to toxicity concerns, technical difficulties, or esthetic considerations. Topical immunomodulators such as imiquimod, a toll-like receptor 7 agonist, have been proposed as alternative therapies in selected cases.^4^^,^^5^ In this manuscript, we report a case of localized facial CL treated successfully with topical imiquimod and monitored noninvasively using RCM, highlighting its potential both as a therapeutic option and as a diagnostic-therapeutic monitoring tool.

## Materials and methods

For the case described herein, we collected pertinent demographic and clinical data, including gender, age, and relevant personal medical history. Dermoscopic images were acquired using a Vidix 4.0 videodermatoscope (Canfield Scientific Inc), at 20× magnification. RCM evaluation was performed using a VivaScope 1500 system (Caliber Imaging & Diagnostics) at baseline (prior to therapy) and 4 weeks after the end of treatment in order to document microscopic features of the lesion and monitor therapeutic response. RCM imaging included mosaics and sequential optical sections obtained from the epidermis to the superficial dermis. Additionally, a comprehensive histopathological description of the diagnostic skin biopsy specimen was provided. The patient was treated with topical 5% imiquimod cream, applied 5 times per week for 4 consecutive weeks, with regular clinical monitoring to document local tolerance and response. All clinical, dermoscopic, and confocal images were reviewed by 3 dermatologists experienced in noninvasive imaging techniques (Antonio Di Guardo, Luca Ambrosio, and Luca Fania). Written informed consent was obtained from the patient for all diagnostic and therapeutic procedures, as well as for the publication of anonymized clinical and imaging data, in accordance with institutional ethical standards.

## Results

**Fig 1:**
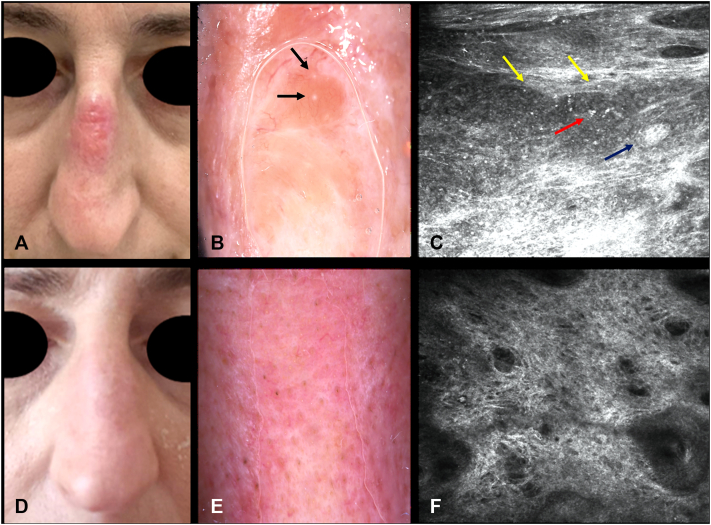
**A,** Clinical presentation of a progressively enlarging erythematous and yellowish plaque located on the dorsal aspect of the nose. **B,** Dermoscopic image of the lesion highlighting an orange structureless area, white scar-like areas, bright “teardrops” (*black arrows*), and arborizing vessels, which are suggestive of granulomatous infiltration. **C,** RCM evaluation revealing clusters of intertwined dendritic Langerhans cells, resembling a nest (*blue arrow*); within the granulomatous structures (*yellow arrows*), multiple bright round cells (*red arrow*) consistent with leishmanial bodies are visible. **D,** Clinical image showing complete resolution of the plaque following 8 weeks of treatment with topical imiquimod. **E,** Corresponding dermoscopic image demonstrating complete resolution of the dermoscopic features 4 weeks after the end of therapy. **F,** Post-treatment RCM confirming the disappearance of the previously described confocal findings. *RCM*, Reflectance confocal microscopy.

We report the case of a 54-year-old Caucasian woman with no significant comorbidities, who developed a progressively enlarging, erythematous, yellowish plaque on the dorsal aspect of her nose. The lesion had previously been misdiagnosed and unsuccessfully treated multiple times, resulting in continuous progression despite multiple therapies. The patient reported a gradual increase in size over several months, associated with mild itching but no pain or systemic symptoms. The patient reported a gradual increase in size over several months, associated with mild itching but no pain or systemic symptoms. Histological examination of a skin biopsy revealed a dense dermal inflammatory infiltrate rich in macrophages, containing intracellular inclusions consistent with Leishmania amastigotes. Given the lesion’s location and the patient's preference to avoid systemic or intralesional therapies, we opted for off-label treatment with topical 5% imiquimod cream. The patient applied the cream 5 times per week for 4 consecutive weeks. As expected, a local inflammatory response developed during the second week, presenting as moderate erythema and mild edema, both of which were well tolerated. No systemic side effects were reported. Complete clinical resolution was observed 4 weeks after the end of treatment, with excellent cosmetic results and no recurrence at 4 months’ follow-up. Notably, RCM was used to monitor the lesion before and after treatment, as the patient declined to undergo a post-treatment biopsy in this cosmetically sensitive area and preferred a noninvasive follow-up approach. Baseline RCM demonstrated, within the papillary dermis, inflammatory infiltrates with clusters of dendritic cells encircling rounded granulomatous aggregates. These aggregates were bordered by interlacing hyper-reflective fibers and dendritic Langerhans cells, yielding a characteristic “bird’s-nest–like” architecture, often juxtaposed to follicles with keratin plugs. Inside the delimited granulomas, numerous bright, round, highly refractile bodies were evident, consistent with intracellular Leishmania amastigotes. A follow-up RCM evaluation, performed 4 weeks after the end of treatment, revealed complete resolution of these features: the dendritic cell clusters and amastigote-like structures were no longer detectable. At the 3-month clinical follow-up, no evidence of recurrence was detected. The clinical, dermoscopic, and confocal microscopy findings before and after therapy are shown in Fig 1.

## Conclusions

Our case contributes to the growing but still limited body of literature supporting the potential utility of imiquimod as monotherapy in localized forms of CL. Imiquimod acts through toll-like receptor 7 agonism, stimulating innate and adaptive immune responses, notably by activating macrophages and promoting nitric oxide–mediated intracellular killing of amastigotes.^5^ While data on its efficacy remain under discussion, particularly in monotherapy settings,^6^, ^7^, ^8^ our report suggests that short-term, intensive topical regimens may be effective in selected patients unwilling or unable to receive systemic or intralesional treatment. Importantly, this case underscores the emerging relevance of noninvasive diagnostic tools in the management of CL. Alongside dermoscopy, which can assist in lesion characterization and differential diagnosis, RCM offers dynamic, in vivo visualization of hallmark microscopic features of leishmaniasis.^9^^,^^10^ In our experience, RCM was valuable in post-treatment monitoring, allowing confirmation of therapeutic response without the need for additional biopsies. Importantly, the ability of RCM to visualize characteristic features of CL, such as dendritic Langerhans cells and intracellular Leishmania amastigotes, also opens the possibility of its use in the prediagnostic setting, potentially guiding clinical suspicion and, in selected cases, reducing the need for invasive diagnostic procedures. These considerations are especially relevant when standard parasitological methods are impractical or when minimizing procedural morbidity is a priority.^3^ It should be noted that RCM enables visualization of the epidermis and upper dermis, with an imaging depth of approximately 200-250 μm. This range includes the papillary and superficial reticular dermis, where the early inflammatory infiltrate and most parasitized macrophages in cutaneous leishmaniasis are typically located.^11^^,^^12^ In later stages, however, granulomatous aggregates often extend into the mid-to-deep reticular dermis (>250-400 μm) and may therefore escape confocal detection. This depth-related limitation should be taken into account when interpreting RCM findings, particularly in more advanced or chronic lesions. The integration of a topical immunomodulatory therapy with high-resolution noninvasive imaging may represent a promising personalized approach, particularly in cases where cosmetic outcome, invasiveness, and patient preference are key considerations. RCM could, in future, reduce reliance on histopathology for both diagnosis and follow-up, especially when classic parasitological methods are not feasible or available.

In conclusion, topical imiquimod 5% may represent a viable treatment option in carefully selected patients with localized CL, particularly in the context of individualized, less invasive management. Moreover, reflectance confocal microscopy is emerging as a valuable tool for both diagnostic support and therapeutic monitoring, offering a noninvasive solution to assess treatment efficacy in real time. However, further prospective studies are warranted to better define the role of imiquimod and RCM in the multidisciplinary management of CL.

## Conflicts of interest

None disclosed.
